# Immobilization of Ionic Liquid on a Covalent Organic Framework for Effectively Catalyzing Cycloaddition of CO_2_ to Epoxides

**DOI:** 10.3390/molecules27196204

**Published:** 2022-09-21

**Authors:** Qianqian Yan, Hao Liang, Shenglin Wang, Hui Hu, Xiaofang Su, Songtao Xiao, Huanjun Xu, Xuechao Jing, Fei Lu, Yanan Gao

**Affiliations:** 1 Key Laboratory of Ministry of Education for Advanced Materials in Tropical Island Resources, Hainan University, No. 58, Renmin Avenue, Haikou 570228, China; 2 China Institute of Atomic Energy, Beijing 102413, China; 3 School of Science, Qiongtai Normal University, Haikou 571127, China; 4 Liaocheng Luxi Polycarbonate Co., Ltd., Liaocheng 252000, China

**Keywords:** covalent organic framework, ionic liquid, carbon dioxide, catalysis, cyclic carbonates

## Abstract

Transforming CO_2_ into value-added chemicals has been an important subject in recent years. The development of a novel heterogeneous catalyst for highly effective CO_2_ conversion still remains a great challenge. As an emerging class of porous organic polymers, covalent organic frameworks (COFs) have exhibited superior potential as catalysts for various chemical reactions, due to their unique structure and properties. In this study, a layered two-dimensional (2D) COF, IM4F-Py-COF, was prepared through a three-component condensation reaction. Benzimidazole moiety, as an ionic liquid precursor, was integrated onto the skeleton of the COF using a benzimidazole-containing building unit. Ionization of the benzimidazole framework was then achieved through quaternization with 1-bromobutane to produce an ionic liquid-immobilized COF, i.e., BMIM4F-Py-COF. The resulting ionic COF shows excellent catalytic activity in promoting the chemical fixation of CO_2_ via reaction with epoxides under solvent-free and co-catalyst-free conditions. High porosity, the one-dimensional (1D) open-channel structure of the COF and the high catalytic activity of ionic liquid may contribute to the excellent catalytic performance. Moreover, the COF catalyst could be reused at least five times without significant loss of its catalytic activity.

## 1. Introduction

Covalent organic frameworks (COFs) are an emerging class of porous crystalline polymers formed through the linkage of organic building units via strong covalent bonds [1,2,3,4]. They feature a large surface area, highly ordered porosity, designable topological structure and easy modification, making them intriguing materials for various applications, such as catalysis [5,6,7,8,9,10,11], gas adsorption/separation [12,13,14,15,16], energy storage [17,18,19] and environmental remediation [20,21,22,23,24]. In the catalysis field, the flexible regulation of the pore environment (i.e., pore size, shape and size distribution) and large numbers of structural topologies offer more possibilities for creating new patterns of catalytic reactivity. In addition, one-dimensional (1D) open channels found in COFs enable the rapid diffusion of substances to promote catalytic reactions. In contrast to traditional porous materials such as activated carbon and zeolites, well-defined catalytic active sites can be spatially separated within the framework, and the number of catalytic sites can be controlled precisely in the desired manner [25,26]. Furthermore, due to the organic nature of COFs, they can be easily modified through either a bottom-up strategy or a post-synthetic modification strategy. As such, the use of COFs as catalysts or catalyst carriers has developed rapidly over recent years. A variety of catalytic active moieties involving metal ions or nanoparticles, as well as a variety of organocatalysts, have been successfully immobilized onto the skeletons of COFs; the resulting COFs exhibited good catalytic performance in many reactions, such as the Heck reaction [27], the Michael addition reaction [28], the Henry reaction [29], Suzuki–Miyaura coupling [6] and the Diels–Alder reaction [30]. In addition, COFs have been used as a multifunctional catalyst for cascade reactions, such as the Heck-epoxidation tandem reaction [31], the oxidation-Knoevenagel cascade reaction [32] and the addition-oxidation cascade reaction [33].

The greenhouse effect is an important cause of global warming; among various greenhouse gases, CO_2_ is the most frequently implicated in global warming, but is also the most abundant carbon feedstock [34]. In recent years, considerable efforts have been devoted to transforming CO_2_ into useful chemicals. Among various value-added chemicals, cyclic carbonates are an important class of chemical products that have been widely used as polar aprotic solvents, electrolytes for lithium-ion batteries, monomers in polymeric materials and fine-chemical intermediates [35,36,37]. To this end, various catalysts have been developed for the highly effective transformation of CO_2_ into cyclic carbonates, including Schiff bases [38], Salen complexes [39] and metalloporphyrins [40]. Moreover, ionic liquids, including ammonium [41,42], phosphonium [43], imidazolium [44,45] and pyridinium salts [46], have been successfully applied to catalyze the cycloaddition of CO_2_ to epoxides. However, as homogeneous catalysts, ionic liquids are difficult to separate from the reaction system, which limits their practical application on a large scale. For this reason, ionic liquids have been immobilized onto different supports, such as porous silicas [47,48], polymers [49] and metal-organic frameworks used as heterogeneous catalysts for easy product separation [50].

The immobilization of ionic liquids on COFs would combine the unique properties of COFs, such as large surface area, controlled porosity, a 1D open-channel structure and the high catalytic activity of ionic liquids, in one material [51,52,53].In addition, confining ionic liquids within a special pore environment may afford chemical reactions with shape-, size-, chemo- or enantio-selectivity. In order to further explore the potential of ionic liquids immobilized on COFs in heterogeneous catalysis, we here describe a post-synthetic strategy for the immobilization of ionic liquids on the channel walls of a 2D COF, IM4F-Py-COF. The resulting ionic liquid-containing COF (BMIM4F-Py-COF) exhibited excellent catalytic performance regarding the cycloaddition of CO_2_ to epoxides. Furthermore, good recyclability was observed for the ionic liquid-immobilized COF catalyst.

## 2. Experimental

### 2.1. Materials

The building units 4,4′,4″,4‴-(Pyrene-1,3,6,8-tetrayl) tetraaniline (PyTTA), 5,6-bis(4-formylbenzyl)-1-methyl-1H-benzimidazole (IM) and 2′3′5′6′-tetrafluoro-[1,1′:4′,1″-terphenyl]-4,4′-dicarbaldehyde (4F) were synthesized in accordance with the reported procedures [54,55]. All starting materials and solvents, unless otherwise specified, were obtained from commercial resources and used without further purification.

### 2.2. Synthesis of IM4F-Py-COF

IM4F-Py-COF was synthesized following the previously reported protocol [56]. In a typical procedure, PyTTA (21.67 mg, 0.04 mmol), 4F (14.32 mg, 0.04 mmol) and IM (13.76 mg, 0.04 mmol), as well as 2 mL of 1,2-dichlorobenzene, were charged into a 10 mL glass ampule vessel. The mixture was sonicated for 10 min, and 0.2 mL of 6.0 M acetic acid was rapidly added. The vessel was flash-frozen in liquid nitrogen and degassed by three freeze–pump–thaw cycles. The internal pressure of the vessel was decreased to below 5 Pa and the vessel was rapidly flame-sealed. The reaction was carried out at 120 °C for 3 days. The precipitate was separated and washed thoroughly with anhydrous THF and acetone, successively, and dried at 100 °C overnight under vacuum to produce a yellow powder in a 78% yield. Elemental analysis: for C_82_H_48_F_4_N_6_: Calcd. C, 82.52%; H, 4.03%; N, 7.04%. Found: C, 76.11%; H, 4.75%; N, 6.05%.

### 2.3. Synthesis of BMIM4F-Py-COF

The ionization of IM4F-Py-COF into BMIM4F-Py-COF was achieved through a quaternization process. To a 50 mL round-bottom flask were added 50 mg of IM4F-Py-COF, 5 mL of 1-bromobutane and 20 mL of acetonitrile. The reaction was heated under reflux at 80 °C for 24 h. After cooling to room temperature, the precipitate was collected by filtration and washed thoroughly with anhydrous ethanol and acetone, successively. The powder was dried at 100 °C overnight under vacuum to give a dark yellow product in a 95% yield.

### 2.4. General Procedures for Cycloaddition of CO_2_ with Epoxides

The reactions were carried out in a 25 mL sealed Teflon-lined autoclave. Firstly, 3.8 mmol epoxide and 20 mg BMIM4F-Py-COF were charged into the reactor without solvent. The air in the autoclave was then removed by a CO_2_ purge. The autoclave was pressurized up to a desired pressure (generally 4.0 MPa) with CO_2_ and the temperature was raised to 110 °C. The reaction was conducted for 12 h. After the reaction, a small amount of the resultant reaction mixture was sampled from the autoclave for nuclear magnetic resonance (NMR) analysis in order to quantitatively evaluate the conversion of epoxide. The crude product was filtered and purified using column chromatography. The isolated yield was calculated based on the weight of the obtained product.

### 2.5. Characterization

Power X-ray diffraction (PXRD) measurements were recorded on a PANalytical X’Pert model Pro Multipurpose Diffractometer (Davis, CA, USA) using Cu *K*_α_ radiation at 40 kV and 40 mA. The signals were collected from 2θ of 2.5–40° at 0.03° step scan with exposure time of 10 s per step. Nitrogen sorption isotherms were measured volumetrically at 77 K using a Quantachrome Autosorb-iQ2 analyzer (Quantachrome Instruments, Boynton Beach, FL, USA)with ultra-high-purity gases. The fresh samples were activated at 100 °C for 15 h under high vacuum prior to analysis. The Brunauer–Emmett–Teller (BET) model was used to determine the specific surface areas using desorption branches over *P*/*P*_0_ of 0.01–0.05. In all isotherm plots, closed circles describe adsorption data points and open circles are used to represent desorption data points. The pore size distribution was evaluated by the nonlocal density function theory (NLDFT) method. ^1^H and nuclear magnetic resonance (NMR) spectra were recorded by a Bruker Advance III 400 MHz NMR spectrometer (Bruker BioSpin Corporation, Fällanden, Switzerland). Gas chromatography (GC, Agilent 7890A, Agilent, Palo Alto, CA, USA) equipped with a capillary column (HP-5, 30 m × 0.25 mm) using a flame ionization detector was carried out. The Br^−^ content in the COFs was measured by ion chromatography, which was carried out with a Dionex ICS 1100 instrument with suppressed conductivity detection. Elemental analysis was performed using an organic elemental analyzer (vario MACRO cube, Elementar, Langenselbold, Germany). Fourier-transform infrared (FT-IR) spectra were recorded using KBr pellets on a Bruker model TENSOR 27 spectrophotometer. Thermogravimetric analysis (TGA, STA449F3, NETZSCH, Selb, Germany) was performed by heating from room temperature to 800 °C at a rate of 10 °C min^−1^ with a N_2_ flow rate of 20 mL min^−1^.

## 3. Results and Discussion

**Scheme 1:**
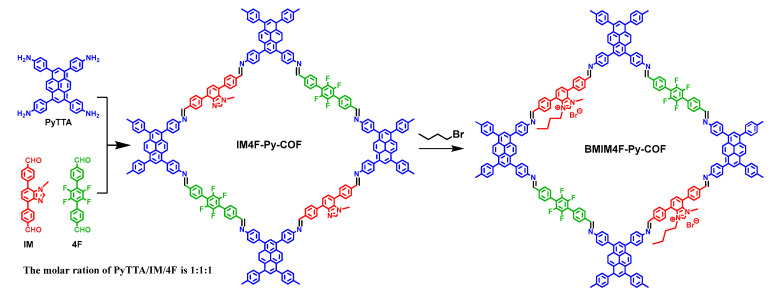
Synthesis procedure of BMIM4F-Py-COF.

The IM4F-Py-COF precursor was first prepared through the condensation reaction of four-branched 4,4′,4″,4‴-(pyrene-1,3,6,8-tetrayl) tetraaniline (PyTTA) and linear 5,6-bis(4-formylbenzyl)-1-methyl-1H-benzimidazole (IM) and 2′3′5′6′-tetrafluoro-[1,1′:4′,1″-terphenyl]-4,4′-dicarbaldehyde (4F) at a molar ratio of 1:1:1 [56]. The fluoro-containing building unit 4F was introduced into the COF to enhance the interlayer interaction that favors the formation of high crystallization and large porosity in COFs [57]. Ionization was achieved through the quaternization reaction with 1-bromobutane to give an ionic COF product, BMIM4F-Py-COF (Scheme 1).

**Figure 1:**
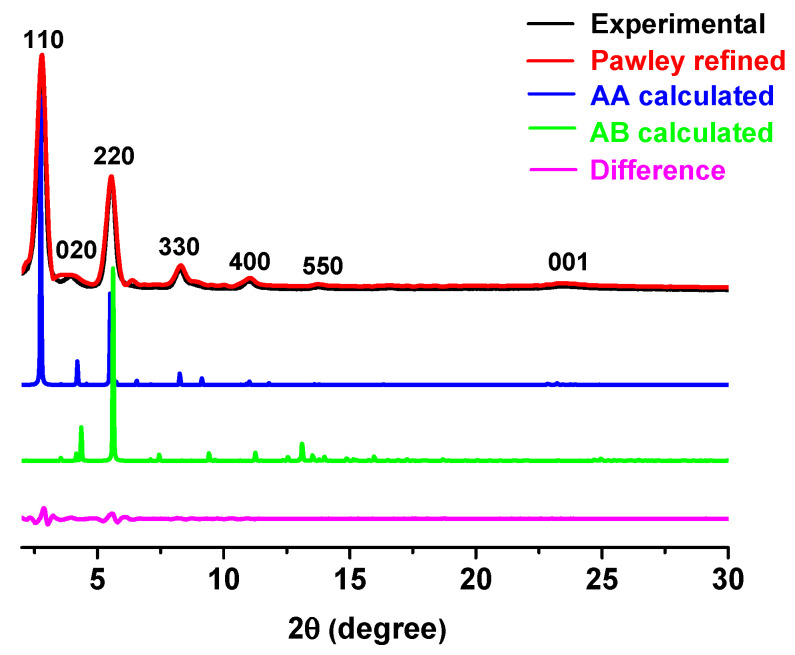
PXRD patterns of IM4F-Py-COF. Experimental pattern (black), profiles simulated using the Pawley refinement (red), the difference between experimental and refined patterns (magenta), AA-stacking (blue) and AB-stacking (green) modes of the IM4F-Py-COF.

The crystallinity of the IM4F-Py-COF was characterized by powder X-ray diffraction (PXRD), as shown in Figure 1. IM4F-Py-COF exhibited several strong diffraction peaks, observed at 2.8°, 3.8°, 5.5°, 8.2°, 10.9°, 13.7° and 23.2°, which can be attributed to the (110), (020), (220), (330), (400), (550) and (001) facets, respectively. The crystalline structure of the COF was analyzed based on the PXRD pattern together with the computational simulation. Given the connectivity and structure of the building blocks, an eclipsed AA stacking model and a staggered AB model were considered. It was found that the AA stacking model reproduced the PXRD pattern well (Figure 1, black, red and blue curves). The final lattice parameters were extracted as *a* = 49.83 Å, *b* = 42.59 Å, *c* = 3.93 Å and *α* = 89.50°, *β* = 88.71°, *γ* = 90.06° after Pawley refinement (Table S1), confirming the peak assignment, as evidenced by the negligible difference (Figure 1, magenta curve). We excluded the possibility of a staggered AB model because the simulated PXRD pattern did not match the observed data (Figure 1, green curve). After the ionization, the produced BMIM4F-Py-COF showed an identical PXRD pattern to that of the precursor IM4F-Py-COF, suggesting that both COFs had similar crystal structures (Figure S1).

**Figure 2:**
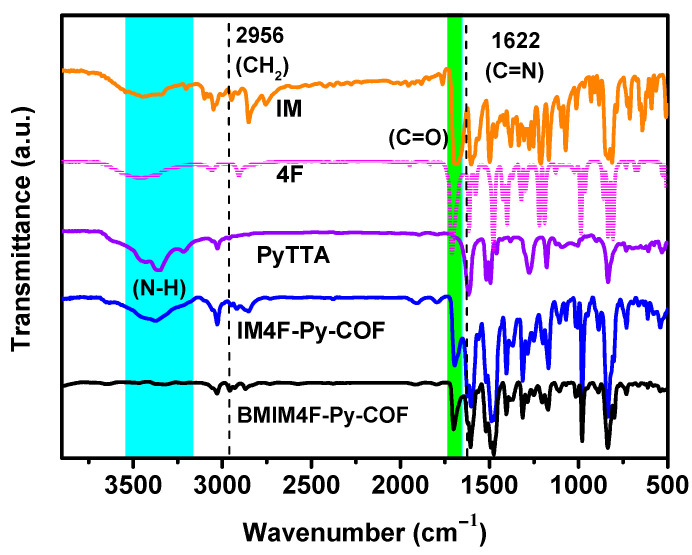
FT-IR spectra of IM4F-Py-COF (blue), BMIM4F-Py-COF (black), IM (orange), 4F (magenta) and PyTTA (violet).

The formation of an imine-linked COF was further confirmed by an FT-IR spectrum (Figure 2). A new peak observed at 1622 cm^−1^ for IM4F-Py-COF and BMIM4F-Py-COF was ascribed to the characteristic peak of the imine (–C=N–) group. In addition, the solid-state ^13^C cross-polarization/magic-angle spinning (CP/MAS) NMR spectrum of IM4F-Py-COF and BMIM4F-Py-COF demonstrated a signal at 150.0 ppm (Figure S2), which was assigned to the carbon atoms of imine linkages, further confirming the imine linkage of the COFs. The ionization of IM4F-Py-COF into BMIM4F-Py-COF was confirmed by the appearance of a new peak at 2956 cm^−1^ in BMIM4F-Py-COF (Figure 2), which was ascribed to the characteristic stretching of –CH_2_–, indicating the successful grafting of n-butyl groups onto the skeleton of the IM4F-Py-COF. Excessive ionization of the COF skeleton would destroy the crystalline structure of the COF, so the quaternization reaction time was set for 24 h. The bromide content in the BMIM4F-Py-COF was measured to be 2.5 wt% (i.e., 0.4 mol%), which means that 42% imidazole moiety was grafted with 1-bromobutane.

**Figure 3:**
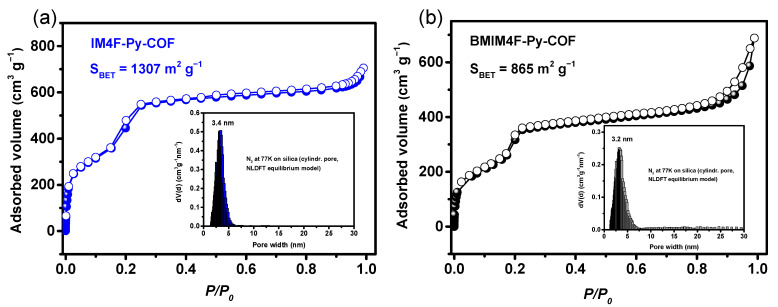
N_2_ sorption isotherms and pore size distribution (insert) of IM4F-Py-COF (**a**) and BMIM4F-Py-COF (**b**).

The porosity of both COFs was evaluated using nitrogen adsorption–desorption isotherms measured at 77 K. The BET surface areas were estimated to be 1307 m^2^ g^−1^, the pore width was calculated to be 3.4 nm and the pore volume was estimated to be 1.09 cm^3^ g^−1^ for IM4F-Py-COF (Figure 3a). After the modification, the BET surface area, the pore width and the pore volume were slightly decreased to 865 m^2^ g^−1^, 3.2 nm and 1.06 cm^3^ g^−1^, respectively (Figure 3b), which was ascribed to the introduction of n-butyl within the pores of the IM4F-Py-COF.

**Figure 4:**
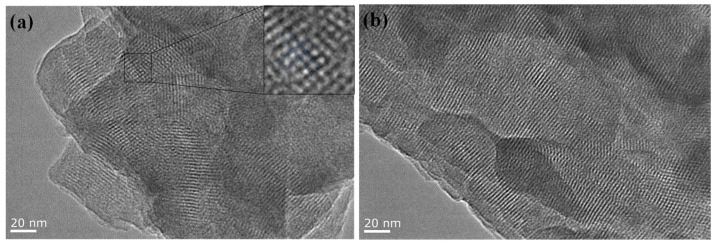
FE-TEM images of (**a**) IM4F-Py-COF and (**b**) BMIM4F-Py-COF. The inset in Figure 4a shows an enlarged image.

The morphology of both COFs was characterized by field-emission scanning electron microscopy (FE-SEM) and high-resolution transmission electron microscopy (HR-TEM). FE-SEM images revealed that IM4F-Py-COF and BMIM4F-Py-COF possessed a lamellar structure several tens of microns in size and with a thickness of hundreds of nanometers (Figure S3). HR-TEM images showed that both COFs had a highly ordered structure, and open channels can be directly observed in Figure 4. Domains oriented along the ab facets perpendicular to the viewing direction showed regular rhombic pores for both COFs (Figure 4a, insert). The results demonstrated the high quality of the COF crystallites. The thermal stabilities of both COFs were also investigated using thermogravimetric analysis (TGA). After modification, the thermal stability of the COF was slightly decreased, although the thermal decomposition temperatures of both COFs were still higher than 400 °C (Figure S4), suggesting their good thermal behavior.

The cycloaddition of CO_2_ to epichlorohydrin was selected as a model reaction in order to establish the activity of the BMIM4F-Py-COF catalyst (Table 1). The reaction mixture was charged in a reactor, which was pressurized with CO_2_ to 4.0 MPa, and reacted at 110 °C for 12 h. Although we aimed to carry out this experiment under milder conditions from an academic perspective, the optimal reaction condition is 4.0–5.0 MPa CO_2_ in practice, due to both material supply and product output issues. Therefore, the CO_2_ pressure was set at the preferred 4.0 MPa in this work. The model reaction produced (chloromethyl)ethylene carbonate in a 97% yield without the use of any solvents (Table 1, entry 1), suggesting an outstanding catalytic performance of BMIM4F-Py-COF. When the amount of BMIM4F-Py-COF catalyst was reduced to half, the yield decreased to 88% (Table 1, entry 2). The effect of temperature and pressure on the product yield was also investigated. In order to compare with our previously reported result [58], the reaction time was set at 24 h here. At this stage, when the temperature was increased from 110 to 120 °C, the BMIM4F-Py-COF demonstrated much better catalytic activity. Even when the pressure of CO_2_ was decreased from 4.0 to 1.0 MPa, the yield of (chloromethyl)ethylene carbonate was still higher than 94% (Table 1, entries 3–6), which is comparable with our previous result [58]. When the reaction time was 12 h, a yield of 91% was observed (Table 1, entry 7), revealing the outstanding catalytic performance of BMIM4F-Py-COF. When the reaction temperature was decreased from 110 to 90 °C, the yield decreased remarkably from 97% to 49% (Table 1, entry 8), suggesting that temperature has a dramatic effect on the yield, in accordance with previously reported results [49,59,60]. Decreasing CO_2_ pressure from 4.0 to 3.0 MPa (110 °C) also led to a decreased yield, from 97% to 77% (Table 1, entry 9). The effect of reaction time on the yield was further studied. When the reaction time was shortened from 12 to 10, 8 and 6 h, the yield decreased from 97% to 94%, 92% and 87%, respectively (Table 1, entry 1 and entries 10–12). When IM4F-Py-COF was used to replace BMIM4F-Py-COF, a yield of only 25% yield was obtained (Table 1, entry 13), suggesting that the reaction was catalyzed by imidazolium bromide active moiety on the BMIM4F-Py-COF.

The catalytic activity of BMIM4F-Py-COF in the cycloaddition of CO_2_ to different epoxides was investigated under identical conditions (Table 2). When propylene oxide was used as an epoxide, a yield as high as 100% was observed (Table 2, entry 1), which is even higher than that of epichlorohydrin (Table 2, entry 2), and is also consistent with previous reports [61,62,63,64,65].In addition, the cycloaddition of CO_2_ to 1,2-epoxyhexane, 1,2-epoxyoctane, butyl glycidyl ether, 3,4-epoxy-1-butene and styrene oxide was also observed (Table 2, entry 3–7). The yield was 97%, 87%, 85%, 88% and 80%, respectively. From the results, it seems that BMIM4F-Py-COF was more effective for small-size substrates [49,59,60,61,62,63,64,65]. We believe that the high catalytic activity of the ionic liquid, the large surface area and the 1D channel walls of the COF may contribute to the excellent catalytic ability of BMIM4F-Py-COF. Although it may seem counter-intuitive to compare the catalytic performance of BMIM4F-Py-COF with other reports because the cycloaddition reactions involve several reaction conditions (e.g., reaction temperature, pressure of CO_2_, solvent, reaction time and dosage of catalyst), we have nonetheless listed a summary of the previously reported catalytic performances of the cycloaddition of CO_2_ to epichlorohydrin (Table S2). In line with the data listed in Table S2, we believe that BMIM4F-Py-COF is a valuable heterogeneous catalyst for cycloaddition reactions, especially considering that the reactions were carried out under solvent-free and co-catalyst-free conditions.

**Figure 5:**
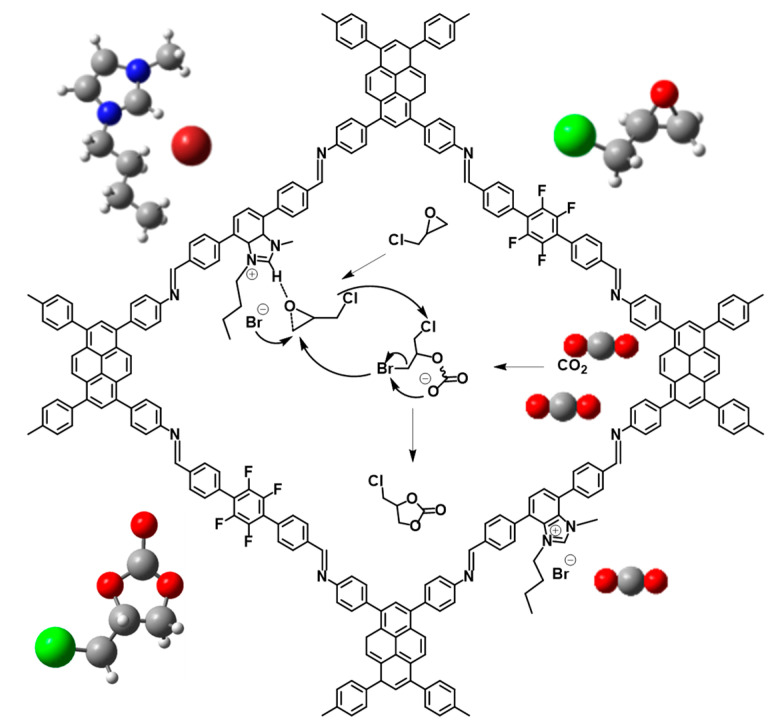
Scheme of possible catalytic mechanism for the reaction of epoxides and CO_2_ into cyclic carbonates catalyzed by BMIM4F-Py-COF.

We consider that the reaction is initiated by binding the O atom of the epoxides with the acidic C_2_-proton of the imidazolium cation, through which process the C-O bond of the epoxides is weakened. Subsequently, the Br^−^ attacks the less-hindered carbon atom of the coordinated epoxide to open the epoxy ring. Subsequently, CO_2_ interacts with the oxygen anion of the opened epoxy ring to form an alkylcarbonate anion, after which a ring closure step gives the cyclic carbonate products (Figure 5) [60,61,62,63,64,65,66].

**Figure 6:**
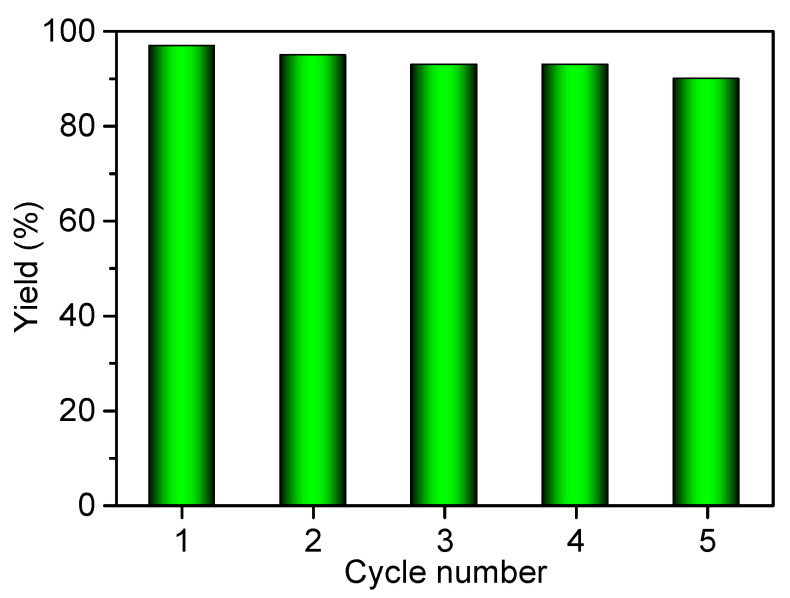
Catalytic activity of recycled BMIM4F-Py-COF for cycloaddition of CO_2_ to epichlorohydrin.

The heterogeneity of the BMIM4F-Py-COF catalyst was investigated by removing the catalyst by centrifugation during an ongoing reaction. Without the catalyst, the conversion stopped, and no significant product formation could be observed. The reusability of the BMIM4F-Py-COF and reproducibility of catalytic performance were investigated based on the experimental results of repeated cyclic tests. In each cycle, BMIM4F-Py-COF was removed by centrifugation and then rinsed with epichlorohydrin. After drying, the catalyst was reused for the next run. The yields of cyclic carbonates in the first four consecutive runs are shown in Figure 6. The results indicated that the catalytic activity of BMIM4F-Py-COF could be retained for up to five cycloaddition series. After five runs, no obvious change was found for the PXRD pattern of BMIM4F-Py-COF before and after the catalysis (Figure S5). In addition, no change was observed for the FT-IR spectrum of BMIM4F-Py-COF after five runs (Figure S6). These results reveal that the BMIM4F-Py-COF can thus be considered a renewable and stable catalyst for the cycloaddition of CO_2_ to epoxides.

## 4. Conclusions

In summary, an ionic BMIM4F-Py-COF was successfully synthesized by grafting an ionic liquid precursor onto the skeleton of a two-dimensional covalent organic framework (2D COF), followed by the ionization of the precursor through the quaternization reaction. The resulting BMIM4F-Py-COF was used as a heterogeneous catalyst for the cycloaddition of CO_2_ to epoxides. It was shown that this ionized porous COF showed good catalytic activity even in a solvent- and co-catalyst-free environment. Furthermore, the BMIM4F-Py-COF was continually recycled five times after easy separation without decreasing its activity or selectivity under equivalent reaction conditions. The high catalytic activity of the ionic liquid, a large surface area and the 1D channel walls of the COF were considered to contribute to the excellent catalytic performance of BMIM4F-Py-COF.

## Figures and Tables

**Table 1:** Influence of various experimental conditions on cycloaddition reaction ^a^.

	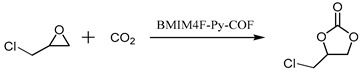						
Entry	Substrate	Catalyst	Pressure	Temperature	Time	Yield ^b^	TON ^c^
			(MPa)	(°C)	(h)	(%)	
1	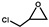	BMIM4F-Py-COF	4.0	110	12	97	589
2 ^d^	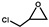	BMIM4F-Py-COF	4.0	110	12	88	1068
3	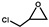	BMIM4F-Py-COF	4.0	120	24	99	601
4	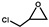	BMIM4F-Py-COF	3.0	120	24	99	601
5	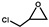	BMIM4F-Py-COF	2.0	120	24	97	589
6	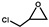	BMIM4F-Py-COF	1.0	120	24	94	571
7	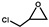	BMIM4F-Py-COF	1.0	120	12	91	553
8	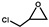	BMIM4F-Py-COF	4.0	90	12	49	298
9	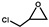	BMIM4F-Py-COF	3.0	110	12	77	467
10	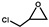	BMIM4F-Py-COF	4.0	110	10	94	571
11	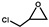	BMIM4F-Py-COF	4.0	110	8	92	558
12	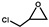	BMIM4F-Py-COF	4.0	110	6	87	528
13	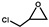	IM4F-Py-COF	4.0	110	12	25	152

^a^ Reaction conditions: epoxide (3.8 mmol), BMIM4F-Py-COF (20 mg, ion content: 0.006 mmol), no additional solvent. ^b^ Product yield was analyzed using gas chromatography (GC). ^c^ TON: moles of synthesized cyclic carbonate per mole of imidazolium salt. ^d^ 10 mg BMIM4F-Py-COF was used as a catalyst.

**Table 2:** Cycloaddition reactions of CO_2_ with various epoxides using BMIM4F-Py-COF as catalyst ^a^.

	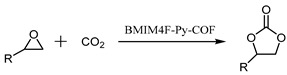						
Entry	Substrate	Catalyst	Pressure	Temperature	Time	Yield ^b^	TON ^c^
			(MPa)	(°C)	(h)	(%)	
1	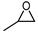	BMIM4F-Py-COF	4.0	110	12	100	608
2	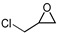	BMIM4F-Py-COF	4.0	110	12	97	589
3	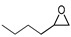	BMIM4F-Py-COF	4.0	90	12	97	589
4	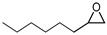	BMIM4F-Py-COF	4.0	110	12	87	529
5	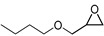	BMIM4F-Py-COF	4.0	110	12	85	517
6	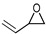	BMIM4F-Py-COF	4.0	110	12	88	535
7	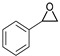	BMIM4F-Py-COF	4.0	110	12	80	486

^a^ Reaction conditions: epoxide (3.8 mmol), BMIM4F-Py-COF (20 mg, ion content: 0.006 mmol), no additional solvent. ^b^ Product yield was analyzed using gas chromatography (GC). ^c^ TON: moles of synthesized cyclic carbonate per mole of imidazolium salt.

## Data Availability

Not applicable.

## Supplementary Materials

The following supporting information can be downloaded at: https://www.mdpi.com/article/10.3390/molecules27196204/s1, Figure S1: PXRD pattern of BMIM4F-Py-COF. Experimental pattern (black) and AA-stacking (blue); Figure S2: Solid-state ^13^C NMR spectra of IM4F-Py-COF (blue) and BMIM4F-Py-COF (black); Figure S3: FE-SEM images of IM4F-Py-COF (a,b) and BMIM4F-Py-COF (c,d); Figure S4: Thermogravimetric analysis of IM4F-Py-COF (blue) and BMIM4F-Py-COF (black); Figure S5: Comparison of PXRD pattern of BMIM4F-Py-COF before (black) and after catalysis (red). The insert indicates an enlarged PXRD pattern; Figure S6: Comparison of FT-IR spectroscopy of BMIM4F-Py-COF before (black) and after catalysis (red); Table S1: Fractional atomic coordinates for the unit cell of IM4F-Py-COF; References citation of [55,56]. Table S2: Comparison with various metal-free catalysts in the performance of the cycloaddition of CO_2_ to epichlorohydrin. References [58,67,68,69,70,71,72,73,74,75,76,77,78,79,80,81,82,83,84,85,86,87] are cited in the supplementary materials.

## Sample Availability

Samples of the building units, IM4F-Py-COF and BMIM4F-Py-COF... are available from the authors.
