# Supplementary material for: Immobilization of Ionic Liquid on a Covalent Organic Framework for Effectively Catalyzing Cycloaddition of CO2 to Epoxides
Source: Molecules. 2022 Sep 21;27(19):6204. doi: 10.3390/molecules27196204 (PMC9570866; doi:10.3390/molecules27196204)
Supplement: Supplementary file 1 [file molecules-27-06204-s001.zip › molecules-1902149-supplementary.pdf]

Supplementary Materials

Immobilization of Ionic Liquid on a Covalent Organic Framework for Effectively Catalyzing Cycloaddition of CO_2 to Epoxides

Qianqian Yan [1,†], Hao Liang [1,†], Shenglin Wang [1], Hui Hu [1], Xiaofang Su [1,*], Songtao Xiao [2,*], Huanjun Xu [3],
Xuechao Jing [4], Fei Lu [1] and Yanan Gao [1,*]

1 Key Laboratory of Ministry of Education for Advanced Materials in Tropical Island Resources, Hainan University, No. 58, Renmin Avenue, Haikou 570228, China
2 China Institute of Atomic Energy, Beijing 102413, China
3 School of Science, Qiongtai Normal University, Haikou 571127, China
4 Liaocheng Luxi Polycarbonate Co., Ltd., Liaocheng 252000, China
* Correspondence: sxf@hainanu.edu.cn (X.S.); xiao200112@163.com (S.X.); ygao@hainanu.edu.cn (Y.G.)
† These authors contributed equally to this work.

Synthesis of 4,4′,4″,4‴-(Pyrene-1,3,6,8-tetrayl) tetraaniline (PyTTA)

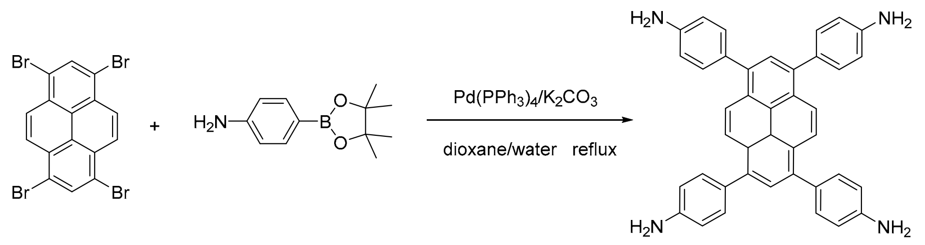

PyTTA was synthesized according to a literature method with modification [54]. To a 250 mL three-necked round flask were added 1,3,6,8-tetrabromopyrene (1.48 g, 2.86 mmol), 4-(4,4,5,5-tetramethyl-1,3,2-dioxaborolan-2-yl)aniline (3.01 g, 13.7 mmol), palladium tetrakis(triphenylphosphine) (0.33 g, 0.29 mmol), and potassium carbonate (2.18 g, 15.7 mmol). The air in the flask was slowly removed by N_2 flow and a mixture of dioxane (32 mL) and H_2O (8 mL) was added to the flask. The reaction was stirred under reflux (115 °C) under N_2 protection for 3 days. After cooling to room temperature, 25 mL water was added to the system and the solid was collected by filtration, and washed thoroughly with H_2O until the filtrate was colorless. The obtained solid was further washed thoroughly with methanol to afford PyTTA in 74% yield. ^{1}H NMR (400 MHz, DMSO) δ 8.14 (s, 4H), 7.80 (s, 2H), 7.35 (d, *J* = 8.3 Hz, 8H), 6.78 (d, *J* = 8.4 Hz, 8H), 5.32 (s, 8H).

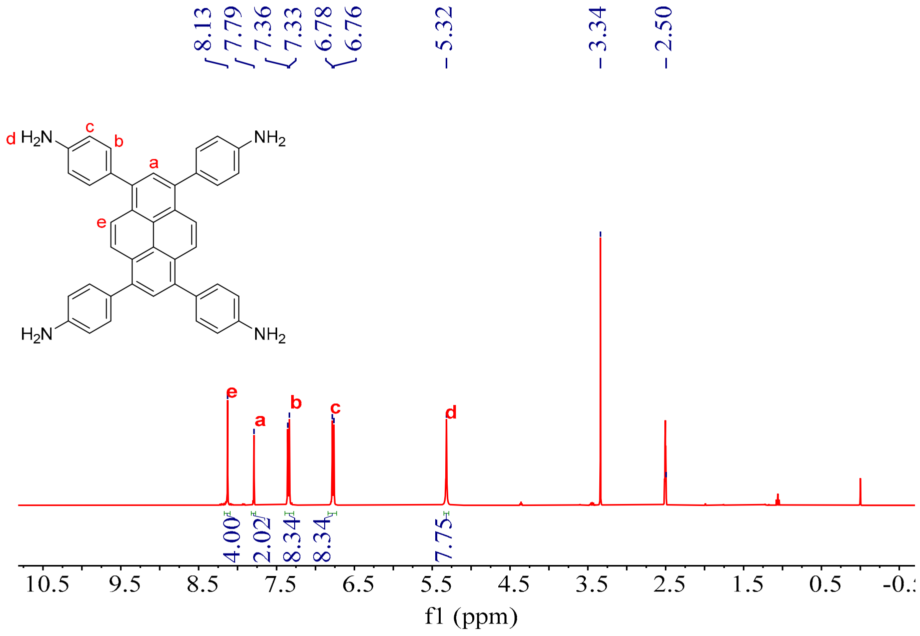

Synthesis of 5,6-bis(4-formylbenzyl)-1-methyl-1H-benzimidazole (IM)

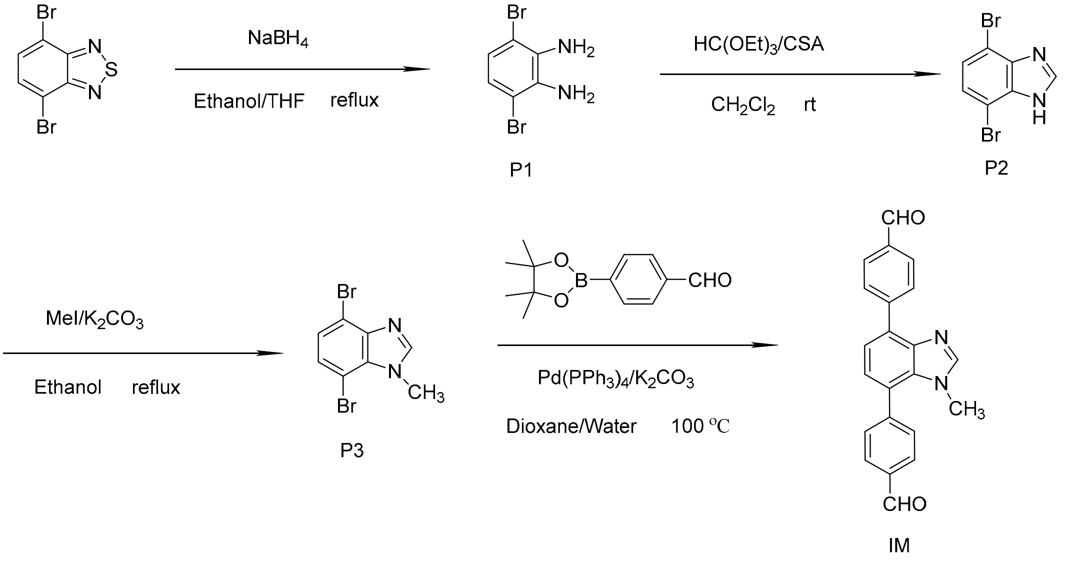

4,5-Dibromo-1,2-benzenediamine. (P1). To a 500 mL two-neck round-bottom flask were added 4,7-dibromobenzothiadiazole (3.5 g, 11.9 mmol), anhydrous THF (40 mL) and anhydrous EtOH (400 mL) under N_2 protection. The mixture was cooled down to 0 °C with vigorous stirring. $NaBH_4$ (2.65 g, 70 mmol for each portion) was added three times (total 8.0 g) every hour. (Be careful! Add slowly. Exothermic nature of the reaction tends to cause bumping. Gas evolution was also observed.) After consumption of 4,7-dibromobenzothiadiazole was confirmed by TLC analysis, the temperature of the mixture was cooled to 0 °C. After slow addition of water (100 mL) and vigorous stirring for 10 min, gummy precipitate was filtered off using Celite. Organic solvent was evaporated and product was extracted with dichloromethane three times. Combined organic layer was washed with water and brine and dried over $MgSO_4$. The extract was filtered off, evaporated, and the crude mixture was purified with short pad silica gel chromatography (eluent: hexane/acetone = 5/1). Combined solution was evaporated to give **P1** as an orange solid in 86.3% yield.

^{1}H NMR (400 MHz, $CDCl_3$) δ 6.85 (s, 2H), 3.74 (s, 4H).

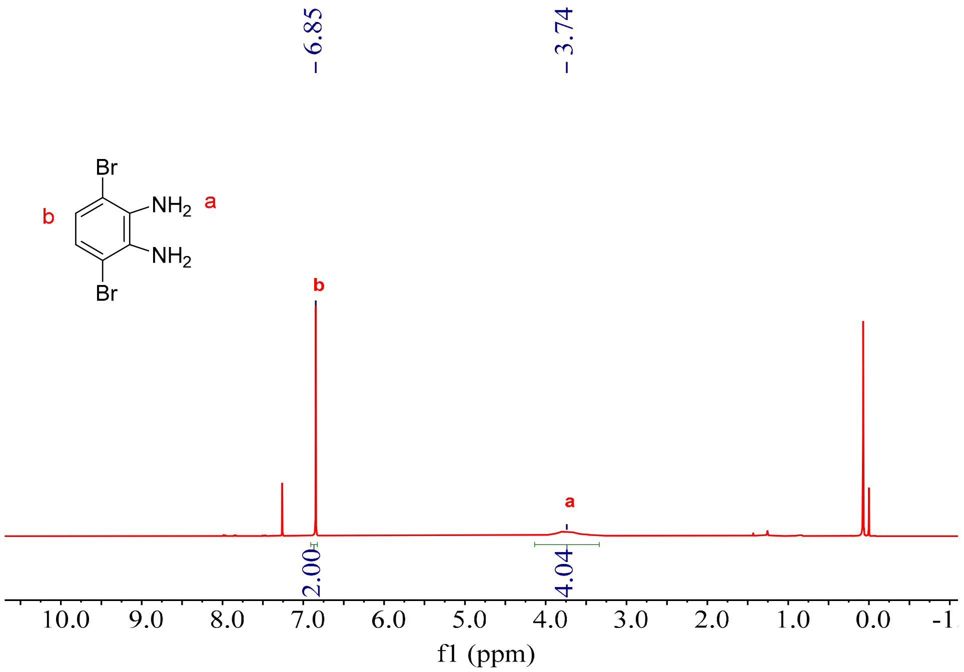

5,6-Dibromobenzimidazole (P2). The newly prepared diamine **P1** (2.16g, 8.11 mol) was immediately used for the next step. To the **P1** dissolved in CH_2Cl_2 (90 mL) were added $HC(OEt)_3$ (1.35 mL, 12.39 mmol) and sulfamic acid (27.5 mg). The mixture was stirred for 3 hours and powder precipitate formed. Solvent was evaporated and the residue was rinsed with ether. After drying under air, P**2** was obtained as a white powder in 67% yield.

^{1}H NMR (400 MHz, DMSO-d_6) δ 8.41 (s, 1H), 7.38 (s, 2H).

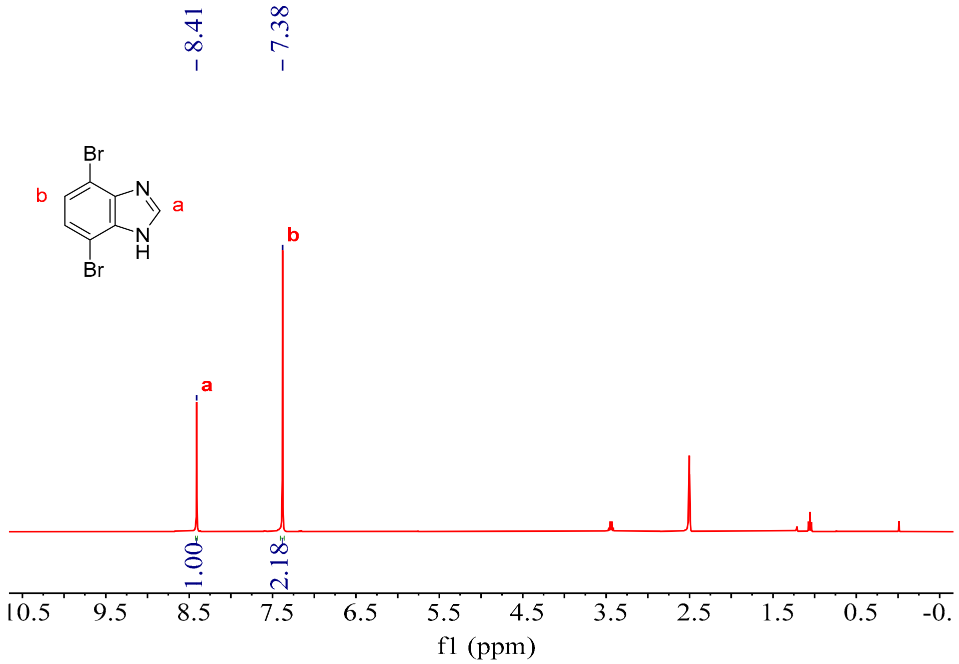

5,6-Dibromo-1-methyl-1*H*-Benzimidazole (P3). To a 250 mL flask were added **P2** (1.97 g, 7.14 mmol), K_2CO_3 (2.96 g, 21.4 mmol) and EtOH (50 mL). The mixture was heated to 50 °C. To the hot mixture, MeI (1.0 mL, 16.2 mmol) was added dropwise and the mixture was maintained at reflux for 3 h. After consumption of **P2** was confirmed by TLC analysis, the mixture was cooled to room temperature. After addition of water (20 mL) and evaporation of EtOH, the powdered precipitate was collected, washed with water and hexane/Et_2O (1/1) successively, and dried under vacuum at 60 °C to produce **P3** as a yellow powder in 86% yield.

^1H NMR (400 MHz, DMSO-d_6) δ 8.36 (s, 1H), 7.37 (s, 2H), 4.09 (s, 3H).

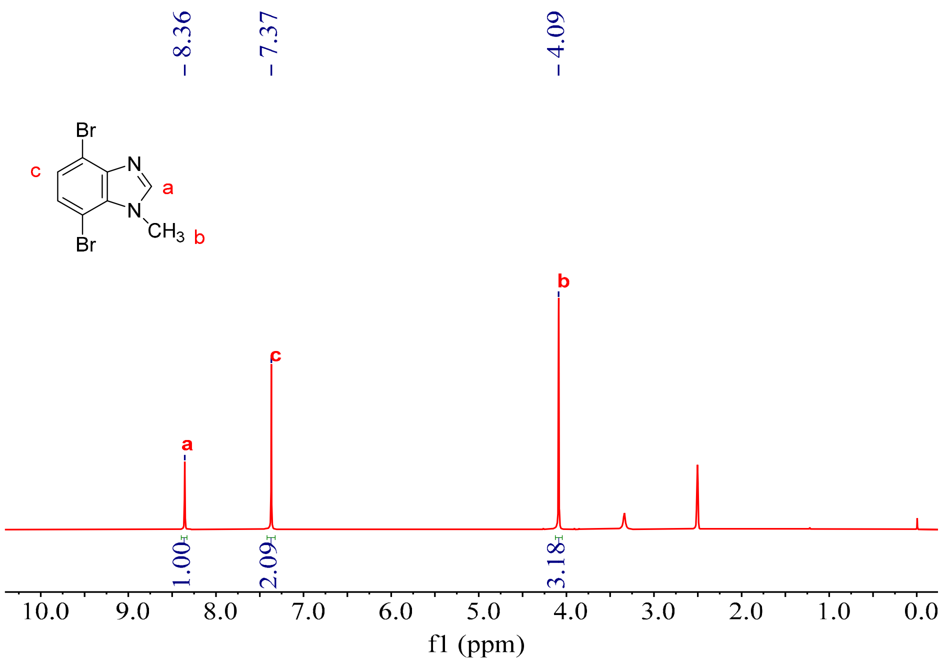

5,6-Bis(4-formylbenzyl)-1-methyl-1*H*-benzimidazole (IM). To a 500 mL three-neck bound bottle flask were added **P3** (1.93 g, 6.67 mmol), 4-(4,4,5,5-tetramethyl-1,3,2-dioxaborolan-2-yl)benzaldehyde (4.67 g, 15.35 mmol), $Pd(PPh_3)_4$ (385 mg, 0.33 mmol) and K_2CO_3 (2.76 g, 20 mmol) in 50 mL of 1,4-dioxane and 12 mL of water with vigorous stirring under N_2 protection. The reaction was heated to 100 ºC overnight under nitrogen atmosphere. The color of the reaction was changed from slight yellow to dark brown. The mixture was cooled to room temperature. Water was added and organic compounds were extracted with ethyl acetate three times. The combined organic layer was washed with brine and dried over anhydrous $MgSO_4$. The extract was

filtered through short pad basic aluminum oxide and evaporated. The obtained residue was rinsed with hexane/Et_2O (2/1) to give **IM** as a brown powder (65% yield).

^{1}H NMR (400 MHz, $CDCl_3$) δ 10.14 (s, 1H), 10.09 (s, 1H), 8.21 (d, *J* = 8.1 Hz, 2H), 8.03 (d, *J* = 11.6 Hz, 4H), 7.93 (s, 1H), 7.67 (d, *J* = 8.0 Hz, 2H), 7.54 (d, *J* = 7.7 Hz, 1H), 7.27 (s, 1H), 3.46 (s, 3H).

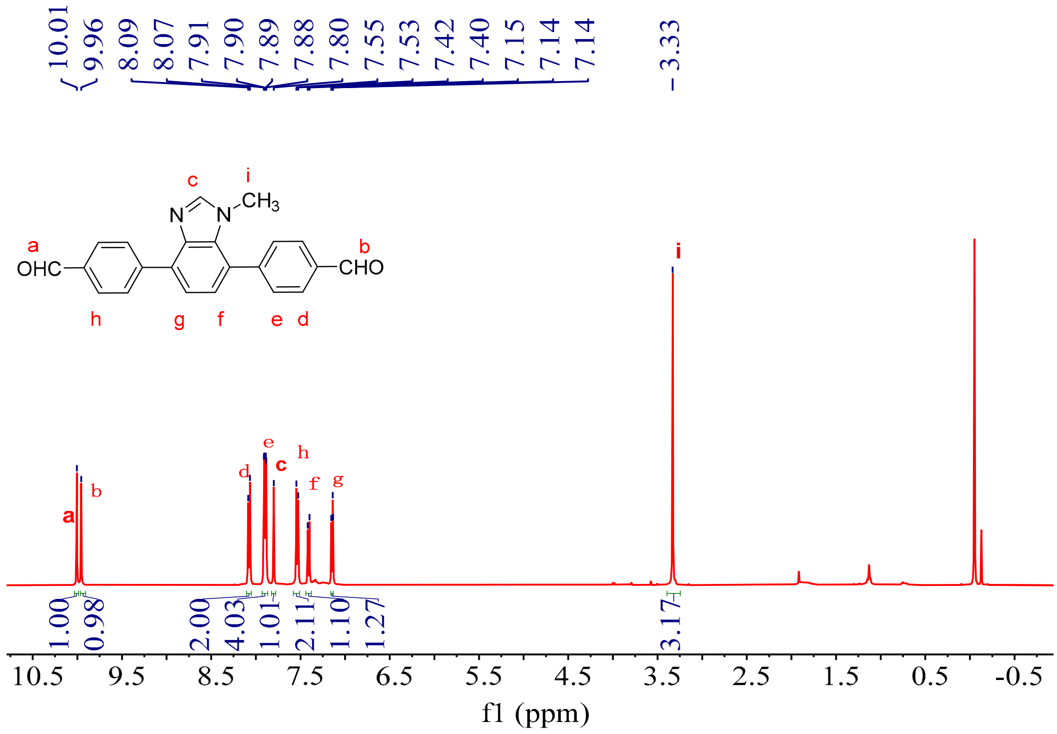

Synthesis of 2′3′5′6′-tetrafluoro-[1,1′:4′,1″-terphenyl]-4,4′-dicarbaldehyde (4F)

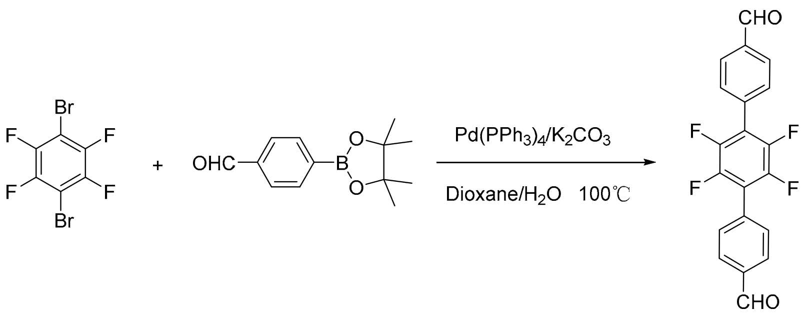

4F was synthesized according to a literature method with modification [55]. To a 500 mL three-neck round bottle flask were added 1,4-dibromotetrafluorobenzene (1.65 g, 5.36 mmol), K_2CO_3 (2.63 g, 19.0 mmol), 4-formylphenylboronic acid pinacol cyclic ester (3.36 g, 14.48 mmol) and palladium tetrakis(triphenylphosphine) (0.35 g, 0.30

mmol). The air in the flask was slowly removed by N_2 flow and a mixture of dioxane (50 mL) and H_2O (12 mL) was added to the flask under N_2 protection. The reaction was stirred under reflux (100 °C) under N_2 environment for 24 hours. After cooling to room temperature, solid was precipitated and separated by filtration. The solid was washed by water, ethyl acetate, and ethanol, successively. After drying at 80 °C under vacuum overnight, a white solid product **4F** was obtained in 80% yield.

^{1}H NMR (400 MHz, $CDCl_3$) δ 10.12 (s, 2H), 8.06 (d, J = 8.4 Hz, 4H), 7.73 (d, J = 8.0 Hz, 4H).

^{13}C NMR (100 MHz, DMSO-d_6) δ 192.87, 136.61, 135.59, 134.04, 132.37, 130.99, 129.79.

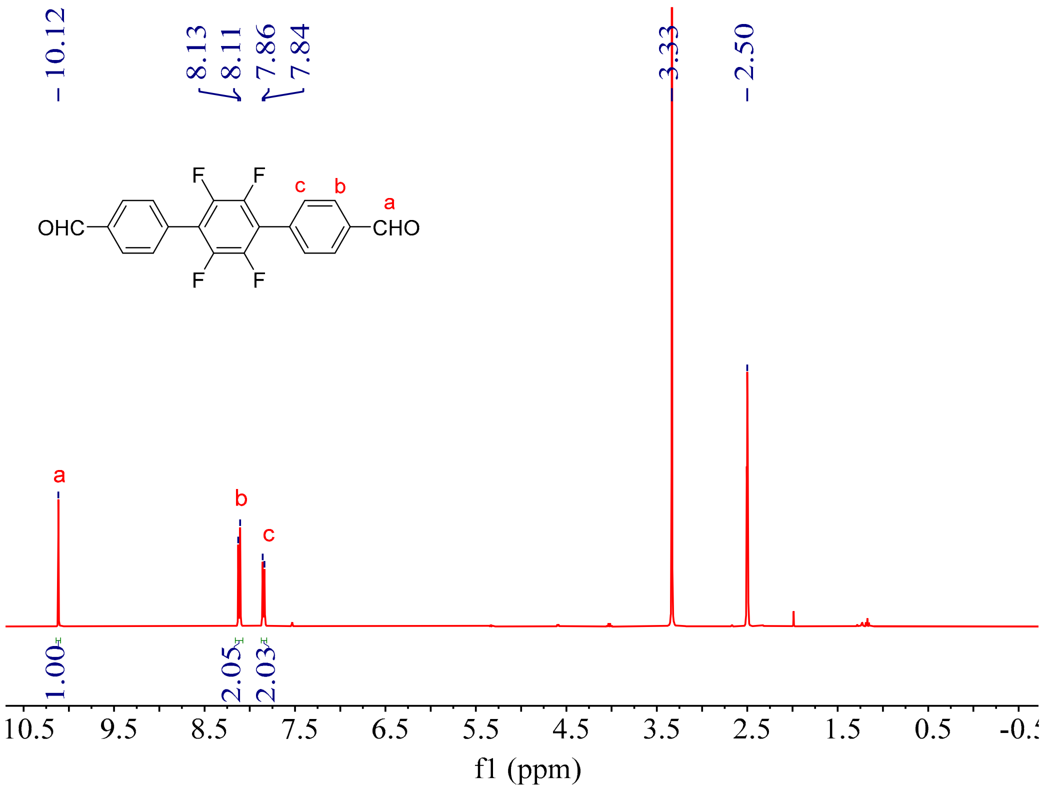

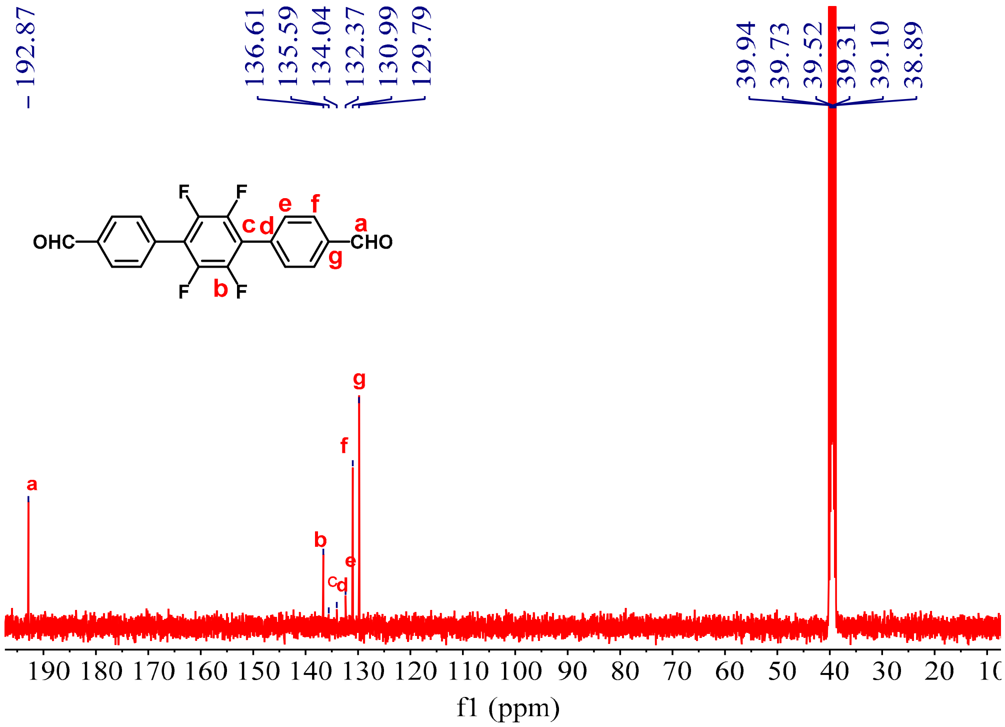

192.87
136.61
135.59
134.04
132.37
130.99
129.79
39.94
39.73
39.52
39.31
39.10
38.89
OHC
CHO
F
a
b
c
d
e
f
g
190 180 170 160 150 140 130 120 110 100 90 80 70 60 50 40 30 20 10
f1 (ppm)

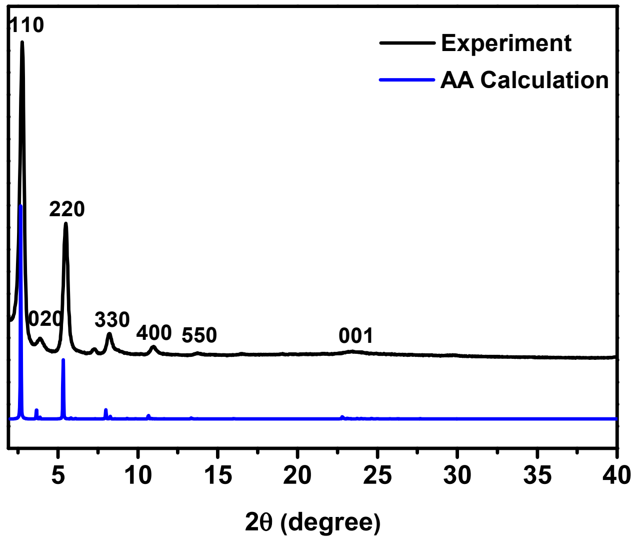

Figure S1. PXRD pattern of BMIM4F-Py-COF. Experimental pattern (black) and AA-stacking (blue).

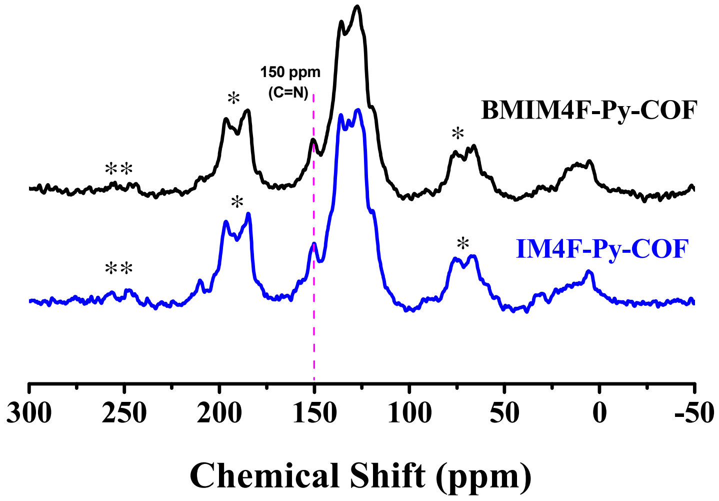

Figure S2. Solid-state ^{13}C NMR spectra of IM4F-Py-COF (blue) and BMIM4F-Py-COF (black).

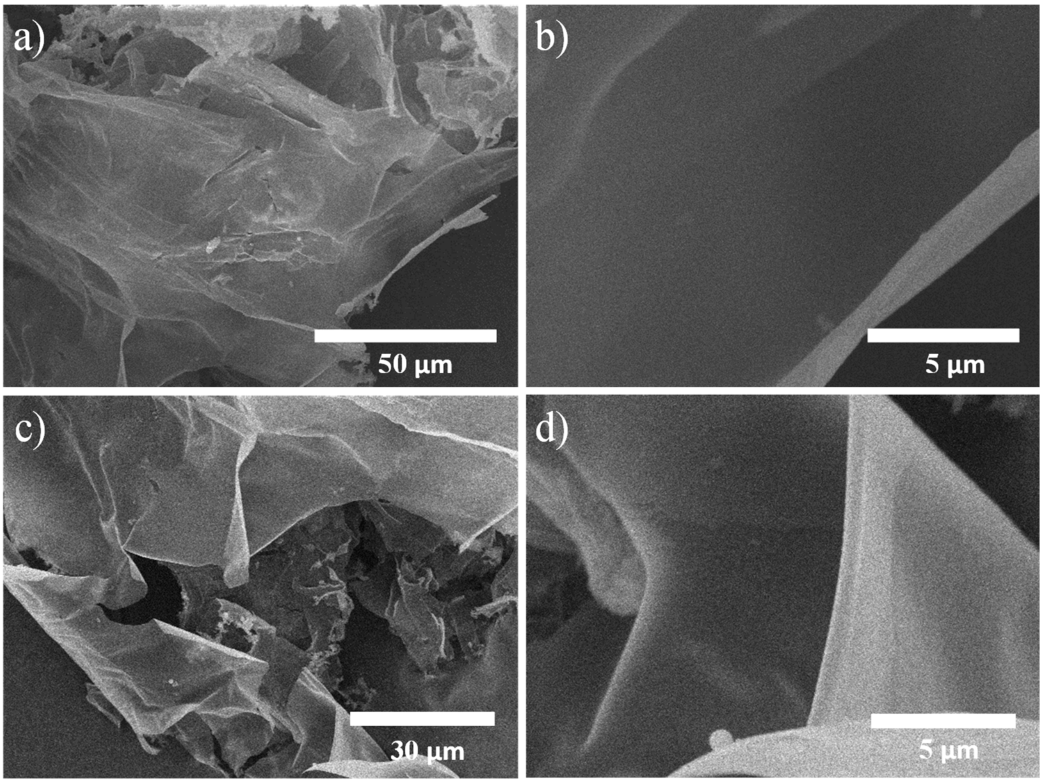

Figure S3. FE-SEM images of IM4F-Py-COF (a,b) and BMIM4F-Py-COF (c,d).

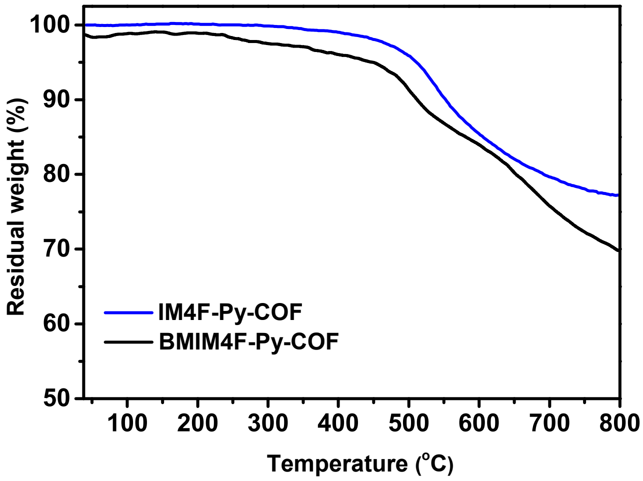

Figure S4. Thermogravimetric analysis of IM4F-Py-COF (blue) and BMIM4F-Py-COF (black).

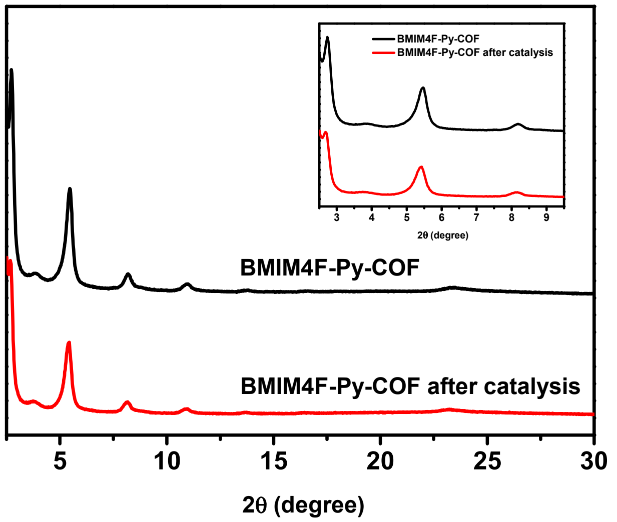

Figure S5. Comparison of PXRD pattern of BMIM4F-Py-COF before (black) and after catalysis (red). The insert indicates an enlarged PXRD pattern.

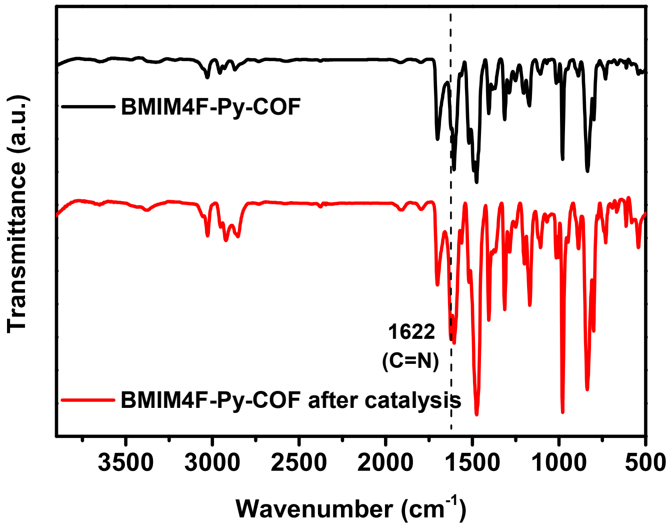

Figure S6. Comparison of FT-IR spectroscopy of BMIM4F-Py-COF before (black) and after catalysis (red).

Table S1. Fractional atomic coordinates for the unit cell of IM4F-Py-COF.

Triclinic P1<br>a = 49.83 Å, b = 42.59 Å, c = 3.93 Å; α = 89.50°, β = 88.71°, γ = 90.06°							
atom	x	y	z	atom	x	y	z
C1	0.17137	0.3325	0.43195	N141	0.87962	0.36	0.4616
C2	0.14814	0.31593	0.53614	C142	0.87981	0.32987	0.4135
C3	0.15022	0.28399	0.62232	H143	0.93161	0.47572	0.68483
C4	0.02362	0.43703	0.52968	H144	0.92779	0.5587	0.18003
C5	0.02361	0.47017	0.56812	H145	0.88675	0.59063	0.17662
C6	0.04726	0.48723	0.62198	H146	0.92489	0.65984	0.79006
C7	0.04851	0.41739	0.51635	H147	0.965	0.62699	0.82272
C8	0.07119	0.58051	0.3033	C148	0.801	0.72269	0.47991
C9	0.09442	0.59894	0.29028	C149	0.77481	0.73897	0.48389
C10	0.09539	0.62757	0.45996	C150	0.7716	0.76734	0.65831
C11	0.0726	0.63759	0.64678	C151	0.82474	0.73929	0.40317
C12	0.04962	0.6187	0.6681	C152	0.80236	0.6905	0.55485
N13	0.11904	0.36079	0.46951	C153	0.75296	0.72597	0.31604
C14	0.12173	0.33153	0.55973	H154	0.86764	0.73715	0.34334
H15	0.06612	0.47574	0.66753	H155	0.82777	0.65022	0.61293
H16	0.0707	0.55927	0.15606	H156	0.82371	0.76418	0.34184
H17	0.11167	0.59141	0.14001	H157	0.78404	0.67764	0.61843
H18	0.07254	0.65927	0.78621	F158	0.79243	0.77809	0.84612
H19	0.03275	0.6264	0.82471	F159	0.75712	0.69979	0.123
C20	0.20237	0.71582	0.45558	H160	0.89856	0.31772	0.36227
C21	0.22924	0.73047	0.42588	C161	0.33118	0.82322	0.56917
C22	0.23254	0.76111	0.29569	C162	0.35451	0.812	0.40498
C23	0.18075	0.73222	0.60411	C163	0.35362	0.78329	0.23423
C24	0.19823	0.68528	0.3364	C164	0.4743	0.93698	0.53173
C25	0.25221	0.71458	0.52995	C165	0.47428	0.97013	0.57209
H26	0.13919	0.73095	0.75928	C166	0.45056	0.98719	0.63263
H27	0.17009	0.64774	0.27165	C167	0.44939	0.91731	0.51651
H28	0.18363	0.75568	0.70077	C168	0.42721	0.08017	0.3216
H29	0.21461	0.67247	0.21651	C169	0.40395	0.09846	0.31476
H30	0.21535	0.77422	0.20861	C170	0.40304	0.12756	0.47483
N31	0.25304	0.68509	0.70083	C171	0.42551	0.13795	0.65137
H32	0.10469	0.31782	0.64928	C172	0.4484	0.11907	0.67041
C33	0.67137	0.8325	0.43461	N173	0.37961	0.86001	0.45914
C34	0.64814	0.81594	0.53872	C174	0.3798	0.82988	0.4109
C35	0.65021	0.78401	0.62512	H175	0.43159	0.97572	0.68322
C36	0.52361	0.93703	0.52961	H176	0.42782	0.05869	0.17848
C37	0.5236	0.97017	0.56803	H177	0.38677	0.09062	0.17448
C38	0.54724	0.98722	0.62228	H178	0.42487	0.15986	0.78801

C39	0.5485	0.91739	0.51677
C40	0.5712	0.08051	0.30392
C41	0.59444	0.09894	0.29131
C42	0.5954	0.12756	0.4612
C43	0.57259	0.13757	0.64777
C44	0.5496	0.11869	0.66867
N45	0.61904	0.86078	0.4713
C46	0.62173	0.83154	0.56194
H47	0.56609	0.97573	0.66813
H48	0.57073	0.05927	0.15657
H49	0.6117	0.09141	0.14123
H50	0.57252	0.15925	0.7873
H51	0.53273	0.12637	0.82507
C52	0.70239	0.21578	0.45857
C53	0.72925	0.23045	0.42901
C54	0.73255	0.26109	0.29888
C55	0.68076	0.23218	0.60705
C56	0.69825	0.18525	0.33922
C57	0.75222	0.21456	0.53309
H58	0.63919	0.2309	0.76193
H59	0.67011	0.14772	0.27405
H60	0.68363	0.25564	0.70385
H61	0.71464	0.17245	0.21937
H62	0.71536	0.2742	0.21179
N63	0.75305	0.18506	0.7039
H64	0.60469	0.81784	0.65155
C65	0.8271	0.67504	0.55202
C66	0.8509	0.69166	0.47652
C67	0.84955	0.72391	0.40336
C68	0.97441	0.5701	0.52606
C69	0.97435	0.53692	0.56979
C70	0.95061	0.5198	0.63267
C71	0.9495	0.58969	0.50977
C72	0.92706	0.42687	0.33047
C73	0.90398	0.40821	0.32115
C74	0.90342	0.37872	0.47833
C75	0.92585	0.36839	0.65649
C76	0.94847	0.38766	0.68004
N77	0.87865	0.64533	0.46801
C78	0.87718	0.67576	0.47755
H79	0.93166	0.53122	0.68294
H80	0.92754	0.44859	0.18761
H81	0.88682	0.41599	0.17916

H179	0.46498	0.12701	0.82132
C180	0.30101	0.22269	0.47673
C181	0.27482	0.23897	0.48069
C182	0.27161	0.26734	0.65513
C183	0.32474	0.23928	0.40006
C184	0.30236	0.19049	0.55172
C185	0.25296	0.22597	0.31284
H186	0.36764	0.23714	0.34046
H187	0.32776	0.15021	0.60998
H188	0.32372	0.26418	0.33872
H189	0.28404	0.17763	0.61524
F190	0.29244	0.27809	0.84296
F191	0.25712	0.19979	0.11979
H192	0.39855	0.81773	0.35978
C193	0.17289	0.67127	0.36706
C194	0.15133	0.68751	0.52017
C195	0.15547	0.7181	0.63811
C196	0.02373	0.5701	0.52077
C197	0.02366	0.53693	0.56371
C198	0.04728	0.51984	0.61961
C199	0.0487	0.58966	0.50033
C200	0.07119	0.42631	0.31945
C201	0.09424	0.4076	0.31185
C202	0.09475	0.37889	0.48584
C203	0.07188	0.36931	0.67568
C204	0.0491	0.38844	0.69157
N205	0.11999	0.6452	0.4373
C206	0.12459	0.67281	0.56208
H207	0.06617	0.5313	0.66325
H208	0.07098	0.44754	0.16758
H209	0.11165	0.41487	0.16121
H210	0.07178	0.34788	0.82396
H211	0.03214	0.38104	0.84992
C212	0.19829	0.28551	0.50525
C213	0.22463	0.26964	0.49476
C214	0.22781	0.24126	0.32131
C215	0.1751	0.26886	0.60505
C216	0.19628	0.31739	0.41829
C217	0.2465	0.28275	0.6611
H218	0.13263	0.27077	0.70445
H219	0.17019	0.35711	0.36281
H220	0.17663	0.24415	0.67304
H221	0.21422	0.3303	0.33899

H82	0.92536	0.34598	0.78806
H83	0.96504	0.37966	0.83173
C84	0.80675	0.27639	0.40703
C85	0.78111	0.25885	0.39295
C86	0.77746	0.22746	0.50749
C87	0.82965	0.26625	0.22791
C88	0.80756	0.30545	0.57337
C89	0.75809	0.27514	0.28352
H90	0.87118	0.27465	0.10008
H91	0.83156	0.3453	0.70636
H92	0.82869	0.24544	0.07622
H93	0.78991	0.31429	0.70617
N94	0.79576	0.20568	0.65962
H95	0.75985	0.29907	0.19008
H96	0.89525	0.68994	0.47427
C97	0.3271	0.17504	0.54902
C98	0.35091	0.19165	0.47363
C99	0.34956	0.22391	0.4004
C100	0.47441	0.0701	0.52509
C101	0.47434	0.03692	0.56884
C102	0.45059	0.0198	0.63135
C103	0.4495	0.08969	0.50835
C104	0.42707	0.92688	0.3288
C105	0.40399	0.90822	0.31915
C106	0.40342	0.87873	0.47618
C107	0.42584	0.86838	0.65452
C108	0.44845	0.88765	0.67844
N109	0.37865	0.14533	0.46555
C110	0.37719	0.17576	0.47491
H111	0.43164	0.03121	0.68134
H112	0.42756	0.9486	0.18603
H113	0.38684	0.91601	0.17701
H114	0.42533	0.84597	0.78598
H115	0.46501	0.87965	0.83032
C116	0.30674	0.77642	0.40389
C117	0.2811	0.75888	0.38975
C118	0.27745	0.72748	0.50431
C119	0.32964	0.76628	0.22484
C120	0.30755	0.80548	0.57027
C121	0.25808	0.77516	0.2803
H122	0.37118	0.77467	0.09724
H123	0.33154	0.84532	0.70347
H124	0.32869	0.74546	0.07312

F222	0.20677	0.23018	0.13998
F223	0.24229	0.30897	0.85302
H224	0.10914	0.68565	0.69841
C225	0.67291	0.17124	0.36961
C226	0.65134	0.18748	0.52265
C227	0.65548	0.21806	0.6408
C228	0.52373	0.07009	0.52068
C229	0.52365	0.03692	0.56361
C230	0.54727	0.01983	0.6199
C231	0.5487	0.08965	0.50071
C232	0.5712	0.9263	0.32012
C233	0.59425	0.90759	0.31295
C234	0.59474	0.87889	0.48716
C235	0.57185	0.86932	0.67675
C236	0.54908	0.88845	0.69218
N237	0.62	0.14518	0.43897
C238	0.6246	0.17278	0.56421
H239	0.56616	0.03129	0.66384
H240	0.571	0.94752	0.16813
H241	0.61167	0.91485	0.1625
H242	0.57175	0.84789	0.82517
H243	0.5321	0.88105	0.85032
C244	0.69829	0.78552	0.50831
C245	0.72463	0.76965	0.49791
C246	0.72781	0.74126	0.32448
C247	0.6751	0.76887	0.60808
C248	0.69629	0.8174	0.42119
C249	0.7465	0.78275	0.66424
H250	0.63262	0.77079	0.7072
H251	0.6702	0.85712	0.36531
H252	0.67662	0.74416	0.67619
H253	0.71422	0.8303	0.34193
F254	0.70677	0.73018	0.14312
F255	0.74229	0.80898	0.8561
H256	0.60914	0.1856	0.70065
C257	-0.00103	0.48681	0.56589
C258	-0.00098	0.42149	0.4979
H259	-0.00086	0.39658	0.44522
C260	0.49896	0.9868	0.56535
C261	0.49901	0.92149	0.49738
H262	0.49914	0.89657	0.44471
C263	-0.00101	0.52027	0.56403
C264	-0.00087	0.58566	0.48922

H125	0.28989	0.81431	0.70301		H265	-0.00076	0.61062	0.43415
N126	0.29575	0.70571	0.65647		C266	0.49899	0.02027	0.5635
H127	0.25984	0.79909	0.18685		C267	0.49913	0.08566	0.48869
H128	0.39525	0.18994	0.47172		H268	0.49925	0.11062	0.43363
C129	0.83119	0.3232	0.57212		C269	0.27809	0.68088	0.77094
C130	0.85452	0.31198	0.40782		C270	0.32331	0.69877	0.5445
C131	0.85363	0.28327	0.23715		H271	0.28506	0.65977	0.89677
C132	0.9743	0.43698	0.5327		H272	0.32402	0.69423	0.26718
C133	0.97429	0.47013	0.57305		H273	0.33076	0.67734	0.67478
C134	0.95057	0.48719	0.63396		H274	0.33714	0.71806	0.60658
C135	0.9494	0.41731	0.51794		C275	0.7781	0.18085	0.77401
C136	0.92719	0.58017	0.32324		C276	0.82332	0.19875	0.54764
C137	0.90393	0.59847	0.31674		H277	0.78507	0.15974	0.89979
C138	0.90303	0.62756	0.47696		H278	0.82403	0.1942	0.27033
C139	0.92552	0.63794	0.65331		H279	0.83077	0.17732	0.67796
C140	0.94841	0.61906	0.67199		H280	0.83715	0.21804	0.60967

Table S2. Comparison with various metal-free catalysts in the performance of the cycloaddition of CO_2 to epichlorohydrin.

Catalysts	Temperature (^{o}C)	Pressure (MPa)	Yield (%)	Ref
BMIM4F-Py-COF	110	4	97	This work
BMIM4F-Py-COF	120	1	94	This work
2,3-DhaTph	110	0.1	88	67
CTF-1	130	0.69	77	68
CTF-1-HSA	130	0.69	96	69
cCTF	90	1	95	69
PAD-3	110	1	99	70
DVB-HTA	120	1.2	90	71
DVB-BTA	120	1.2	84	71
POM3-IM	120	1	90	72
PRP-1+TBAB	60	0.1	89	73
[PTPP]50%-TD-COF	100	1	99	74
PIL-HPCOF-320–10–100	90	1	95	75
IP-1	100	0.1	76	76
IP-2	100	0.1	99	76
IP-1	80	0.1	52	76
IP-2	80	0.1	70	76
COF-JLU7/TBAB	80	0.1	96	77
DB10%-Pa-Tp	120	0.1	99	78
COF-IL	80	0.1	98	79
PAIL-1	25	0.5	10	80
PAIL-2	25	0.5	22	80

PAIL-3	25	0.5	41	80
PAIL-3	80	0.5	99	80
CTF-0-400-40-1	130	0.69	<5	81
CTF-0-400/600-20/20-5	130	0.69	93	81
CTF-CSU1	25	0.1	78	82
COF-JLU7	40	0.1	92	77
OMe-OH-TPBP-COF	40	0.1	91	83
COF-salen-Co	100	2	90	84
DB10%-Pa-Tp	120	0.1	99	85
COF-HNU3	100	2	96	86
AMIMBr@H2P-DHPh COF	120	1	91	58
COF-HNU14	120	2	99	87
